# Prevalence estimates of depression and anxiety symptoms among adolescents in Bermuda, according to age, gender and race

**DOI:** 10.1007/s00127-025-02829-z

**Published:** 2025-03-11

**Authors:** Daniel Cavanagh, Laura M. Hart, Shawnee Basden, Shurong Lu, Nicola Reavley

**Affiliations:** 1 Centre for Mental Health and Community Wellbeing, Melbourne School of Population and Global Health, University of Melbourne, Melbourne, Australia; 2 Department of Arts and Science, Bermuda College, Paget, Bermuda

**Keywords:** Depression, Anxiety, PHQ-8, GAD-7, Prevalence, Adolescents

## Abstract

**Purpose:**

Common mental disorders (CMDs) among adolescents, such as anxiety and depression, are associated with significant impairment and have been exacerbated by the COVID-19 pandemic. The Caribbean, including Bermuda, lacks sufficient CMD prevalence data to inform policy and service provision for adolescent mental health. This study sought to estimate the prevalence of depression and anxiety symptoms among adolescents in Bermuda.

**Methods:**

This cross-sectional study surveyed middle and high school students aged 10–18 years in Bermuda. 15 schools participated in data collection. Online surveys conducted between November 2022 - June 2023 gathered demographic data and assessed depression symptoms using the PHQ-8, anxiety symptoms using the GAD-7, and impairment across daily activities, school/work and relationships.

**Results:**

Of a total of 2,526 adolescents in Bermuda who self-reported depression and anxiety symptoms, the estimate prevalence of moderate to severe depression symptoms was 31.3%. Prevalence was significantly higher among older adolescents, females and those that identified as Black or Minority. Among the 25.2% who reported moderate to severe anxiety symptoms, prevalence was significantly higher among older adolescents, females and those who did not identify as Minority. Furthermore, 65.6% of adolescents self-reporting moderate to severe depression symptoms reported comorbid moderate to severe anxiety symptoms. The rates of impairment for depression and anxiety were 22.6% and 19.1%, respectively.

**Conclusion:**

The prevalence of depressive and anxiety symptoms among Bermuda’s adolescents is high, surpassing post-pandemic global averages. Findings improve our understanding of CMDs in the Caribbean and provide direction for improved policy and service provision in Bermuda.

**Supplementary Information:**

The online version contains supplementary material available at 10.1007/s00127-025-02829-z.

## Introduction

Common mental disorders (CMDs) such as anxiety and depression are highly prevalent in adolescent populations globally, relative to other age groups [1]. Indeed, half of all lifetime cases of mental disorders start by age 14 years, with most cases going undetected and untreated [2], leaving depression and anxiety as the leading contributors to the health burden among children and adolescents [1]. Global prevalence estimates are, however, limited by a lack of coverage; compared to the estimated size of the adolescent population, the proportion of adolescents who are represented in the available primary data is low [3]. Increasing coverage of prevalence estimates is vital to drive investment in the prevention and treatment of mental disorders when compared to other diseases of childhood. This particularly applies to regions where there has been little or no primary data collection.

In the Caribbean, the proportion of adolescents represented by the available data is sub-optimal. The mean coverage, defined as the proportion of the target population represented by the available data, of prevalence data for mental disorders for those aged between 5 and 17 years is 7.3%, with 8.2% coverage for depression and 7.3% coverage for anxiety disorders [3]. Many studies do not assess for anxiety and depressive disorders directly (e.g., using clinical interviews), but rather, they use screening tools to estimate the prevalence of symptoms of anxiety and depression. Studies in the Caribbean are typically from English speaking independent nations of the British Commonwealth, such as Jamaica, the Bahamas, St. Kitts and Nevis, and St. Vincent. Studies conducted between 2008 and 2012 suggested high rates of moderate to severe symptoms of depression among adolescents in Grade 10 (24.5–40.7%) [4–8].

The islands of Bermuda are often categorised as a Caribbean UK Overseas Territory (formerly British West Indies), given its English-speaking population whose head of state is the constitutional monarch of the United Kingdom. Since 2003, the Bermudian Department of National Drug Control has conducted five National School Surveys investigating alcohol and substance use. However, no studies have been conducted on anxiety and depression among adolescents. While the Caribbean studies point to the possibility of higher rates of depression and anxiety symptoms among the Bermudian adolescent population, there are a number of important reasons why the Caribbean studies should not be extrapolated to Bermuda.

First, Bermuda, is located in the mid-Atlantic Ocean and neither geologically nor spatially connected to the Caribbean, which lies over 1000 km to the south and southwest. Second, while most English-speaking Caribbean countries are of middle income status, Bermuda has one of the highest incomes in the world with a GDP per capita of $113,755 [9]. Third, Bermuda appears to provide more mental health support services to adolescents compared to other Caribbean UK Overseas Territories. All Caribbean UK Overseas Territories, including Bermuda, have mental health support for adolescents through school counsellors and / or educational psychologists [10]; however, unlike most of them, Bermuda has dedicated mental health services for children and adolescents including inpatient and outpatient services and clinical psychology [11]. Despite these services, a situational analysis of the mental health system of Bermuda reported the desire to increase service provision for this population due to a rising demand from schools and primary care [10]. Fourth, the prevalence studies conducted in the Caribbean were conducted prior to the COVID-19 pandemic, which may have led to an increase in the prevalence of CMDs among adolescents. One meta-analysis of 29 studies including 80,879 youth found that during the first year of the pandemic 1 in 4 youth aged under 18 years were estimated to be experiencing moderate to severe symptoms of depression and 1 in 5 were estimated to experience moderate to severe symptoms of anxiety [12]. This represented a doubling of pre-pandemic estimates. Moreover, these researchers found the increase in the prevalence of depression symptoms during the pandemic was higher for older adolescents in comparison to younger adolescents. Similarly, female adolescents have historically found to have higher prevalence of anxiety disorders compared to males, with ratios estimated between 2:1 to 3:1 [13, 14]. This ratio may have increased during the pandemic as a 2020 systematic review of global prevalence of depression and anxiety disorders found that prevalence rates among females were more impacted by the pandemic than males, as increases in prevalence among females for these disorders outstripped increases in prevalence among males [15].

Finally, Bermuda’s population has a unique racial/ethnic composition. In the 2016 Bermuda Census [16], adolescents aged 10–19 years made up approximately 10% of the total population. Among these adolescents, 54% identified as Black, 25% as White and 20% as Mixed and Other, with the largest minority among the latter category identifying as Portuguese. This suggests there are a smaller proportion of Black adolescents compared to other English speaking Caribbean countries such as Jamaica, the Bahamas and St. Kitts and Nevis, where 91%, 85% and 66% of adolescents respectively identify as Black [4]. The racial composition of Bermuda is important to consider in relation to the prevalence of common mental disorders as there may be important differences across groups. For example, in the USA, although Black Americans are often found to have higher rates of physical illness, poverty and psychosocial stressors than White Americans, they also report similar or better mental health than White Americans [11–13], in particular a lower prevalence of diagnosed depression [14, 15], resulting in what is sometimes called “the Black-White paradox” [16, 17]. Although the paradox is not always found in prevalence studies investigating race [18], it has been found to apply to Caribbean Black adults in the USA [19]. The Black-White paradox also appears to apply to adolescent populations [20], although it is less researched. One study found that Black adolescents born in the USA between 1957 and 1969 reported lower prevalence of anxiety disorders than White adolescents, yet Black adolescents between 1983 and 1991 reported higher prevalence of anxiety disorders [21]. This supports other studies from the US which reported higher prevalence of depression symptoms among Black adolescents [22, 23]. It is questionable whether the Black-White paradox may apply to the Bermudian context, as limited data is available on the range of factors that influence mental health outcomes. In either case, Bermuda’s unique mix of racial and ethnic groups suggests assessing prevalence estimates is particularly important because studies from other countries may not be generalisable.

Ultimately, given the unique context within which Bermudian adolescents live, and the need to better support their mental health after COVID-19, calculating contemporary prevalence estimates for CMD symptoms is an important endeavour. This is especially true if evidence-informed health policies are to be developed and health resources are to be appropriately allocated. The aim of the present study was therefore to estimate the prevalence of depressive and anxiety symptoms among adolescents in Bermuda, and to explore how key demographic variables such as age, gender and race, may be associated with differences in prevalence estimates.

Methods.

### Study population and data collection

This cross-sectional study aimed to survey all adolescent students attending middle and secondary education in Bermuda. According to school records from the 2022–2023 Academic Year the potential participant population was estimated to be 3,593 adolescents aged 10–18 years. Students were recruited through all but one of the 16 middle (grades M1 through M3) and high (Grades S1 through S4) schools in Bermuda, which included eight private schools, six public (government-funded) schools and two government-funded alternative education schools One of the other alternative education school serves students with severe to profound learning disabilities and complex care needs. The inability of students attending this school to provide assent means they were excluded from participation. Additionally, schools with very small sample sizes (home schools) or with students outside the eligible age range (the community college) were excluded. The number of students attending middle and high schools is relatively even between private (51%) and government (49%) institutions.

Online surveys were administered during students’ regular class time under the supervision of a teacher at their school between 3 November 2022 and 1 June 2023. Ethics approval for the study was obtained from the University of Melbourne Human Research Ethics Committee (ID: 23177) and the Bermuda Hospitals Board Institutional Review Board. The study used opt-out parental consent whereby parents/guardians were considered to have consented to their child participating unless they returned a form to their child’s school to indicate they did not want their child to participate (i.e., unless they opted-out). To ensure parents/guardians were aware of the research and the opportunity to opt their child out of the study, an extensive information campaign was conducted by the research team. Student assent was also obtained prior to participation by having students indicate their willingness to participate on the landing page of the survey. A unique Research ID was generated by the research team for each potential participant and schools assigned these to students within their school. Students were asked to enter their Research ID alongside their assent on the survey, as this enabled the school to follow up with students who requested support at the end of the survey, while preserving the anonymity of the students to the research team. Every student that assented to the survey was provided with a resource sheet outlining mental health service providers that could assist with any mental health concerns or queries.

### Measures

The online survey was hosted by the survey platform Qualtrics [24]. It was designed to gather information about students’ demographic characteristics, depression and anxiety symptoms, as well as other mental health literacy and help-seeking components (not reported here).

The survey asked students to indicate their age (by entering their month and year of birth), gender (by selecting ‘Male’, ‘Female’ or ‘I identify with another term’) and race (by selecting ‘Black’, ‘White’, ‘Portuguese’, ‘Mixed’, ‘Asian or Pacific Islander’ or ‘Other’; those who identified as ‘Other’ could specify in an open-ended response).

The PHQ-9 (Patient Health Questionnaire-9), a validated screening tool [25], was used to assess symptoms of depression according to the *Diagnostic and Statistical Manual of Mental Disorders 5th edition* [26] criteria, over the past two weeks. The PHQ-9 has demonstrated validity and reliability in detecting adolescent depression across countries and cultures [27–30]. Participants completed an eight-item version of the instrument (the PHQ-8) which excludes the item relating to suicidality (“*Thoughts that you would be better off dead*,* or of hurting yourself in some way?”*), as per previous studies with adolescents [31, 32]. This item was excluded to maintain the anonymity of participants, given the ethical obligation to intervene if students reported suicidal ideation. Previous research suggests that omission of this item does not lead to any reductions in the validity or reliability of the instrument [33].

The PHQ-8 uses a four-point scale of frequency for responding (*not at all [0]*,* several days* [1], *more than half the days* [2] *nearly every day* [3]*)* and asks participants to identify eight symptoms, such as: ‘little interest or pleasure in doing things’ and ‘feeling down, depressed or hopeless.’ The total PHQ8 score ranges between 0 to 24 with higher scores indicating more severe depression. Cut points for indicating mild, moderate, moderately-severe and severe depression were set at 5, 10, 15 and 20, respectively, in accordance with previous research [25]. Scores of 10 and above were categorised as ‘moderate to severe’ and included in further analyses relating to impairment and comorbidity. With a cut-point score of 10 and above, the sensitivity and specificity of the PHQ-8 is 100% and specificity 95%, respectively, for major depressive disorder [34]. As such, scores of 10 and above were categorised as ‘moderate to severe’ and included in further analyses relating to impairment and comorbidity.

The General Anxiety Disorder– 7 (GAD-7), a validated screening tool [35], was used to assess symptoms of Generalised Anxiety Disorder according to the *Diagnostic and Statistical Manual of Mental Disorders 5th edition* [26] criteria, over the past two weeks. The GAD-7 has demonstrated validity and reliability in detecting adolescent anxiety across countries and cultures [31, 36, 37]. Like the PHQ-8, participants respond to each question on the same four-point scale. Cut points for indicating mild, moderate and severe depression were set at 5, 10 and 15, respectively. The GAD-7 sensitivity and specificity using a cut score of 10 are 89% and 82%, respectively [35]. As with the PHQ-8, scores of 10 and above were categorised as ‘moderate to severe’ and included in further analyses.

Impairment was measured across three domains, as recommended by the UNICEF Measuring Mental Health Among Adolescents and Young People at the Population Level (MMAPP) initiative [38]. After responding to the PHQ-8 and GAD-7, participants were asked how often these problems have interfered with daily activities, school/work and relationships with peers, over the past month. Participants responded on a four-point scale including *never (0)*,* sometimes (1)*,* often (2)* and *always (3)*, with a higher score reflecting a greater degree of impairment. Responses that reported ‘often’ (2) or ‘always’ (3) were collapsed into one category of impairment and included in further analyses.

### Statistical analyses

Demographic variables were summarized as n (%) across the sample. Age was collapsed into two categories– younger adolescents (those aged 10–14 years) and older adolescents (those aged 15–18 years). These age groups roughly map onto the middle versus high-school years. For gender, 1.7% (*N* = 42) of participants reported ‘I identify with another term’ (19 younger adolescents, 20 older adolescents, 3 of unknown age). To ensure this subgroup is included and represented in our reporting, the data of adolescents who reported identifying with a term other than male or female are included in the summary statistics. Due to this small subgroup sample size, however, further analyses were restricted to male and female categories only. Race was collapsed into three categories: Black, White and Minority– where Minority included those who reported as ‘Mixed’ (17.2%), ‘Portuguese’ (6.9%), ‘Asian or Pacific Islander’ (2.1%) or ‘Other’ (2.8%).

The scores for the PHQ-8 and GAD-7 were reported as mean ± standard deviation. For both the PHQ-8 and GAD-7, the severity of symptoms, the presence of moderate to severe symptoms and the presence of moderate to severe symptoms with impairment were all summarised as n (%).

Next, we conducted binary logistic regressions to examine whether the independent variables of age, gender and race were associated with the dependent variables. In the first series of binary logistic regressions the dependent variables were the presence of moderate to severe symptoms depression symptoms with and without impairment in at least one of the following domains: daily activities, school or work, or in relationships with peers. In the second series of binary logistic regressions the dependent variables were the presence of moderate to severe symptoms anxiety symptoms with and without impairment in the same three domains. In the final series of binary logistic regressions the dependent variables were the presence of comorbid symptoms with and without impairment in the same three domains, restricting analyses to those participants reporting moderate to severe symptoms for both depression and anxiety. The following categorical variables were simultaneously entered into the model, where the variable sub-categories that are italicised were the reference categories for each of the respective variables: age (*younger adolescents*, older adolescents), gender (*male*, female) and race (*White*, Black, Minority). Simultaneously entering these variables meant that we could examine the effect of each IV on the DV while adjusting for the effects of the other variables. As such, adjusted odds ratios and their 95% CIs were reported. Statistical analyses were conducted in SPSS (version 29). Only data from participants who assented to the survey and completed all items were included in analyses. Participants who did not complete all PHQ-8 items (*N* = 228), or all GAD-7 items (*N* = 214), were excluded from analyses.

**Table 1:** Demographic characteristics of the sample

Variable	All participants (*N* = 2526)	Females (*N* = 1309)	Males (*N* = 1169)
Age, n (%)			
Younger adolescents	1618 (64)	842 (64)	757 (65)
Older adolescents	887 (35)	460 (35)	407 (35)
Missing	21 (1)	5 (1)	3 (0)
Race, n (%)			
Black	1110 (44)	611 (47)	485 (41)
White	673 (27)	326 (25)	339 (29)
Minority*	733 (29)	369 (28)	343 (29)
Missing	10 (0)	3 (0)	2 (0)
School, n (%)			
Private	1495 (59)	766 (59)	704 (60)
Government	1031 (41)	543 (41)	465 (40)

*Minority accounted for those who identified as ‘Mixed’ (*n* = 435), ‘Portuguese’ (*n* = 174), ‘Asian or Pacific Islander’ (*n* = 53) or ‘Other’ (*n* = 71)

Younger adolescents: 10–14 year olds; Older adolescents: 15–18 years olds

## Results

### Demographic characteristics

A total of 2,526 adolescents in Bermuda assented and completed the survey (female = 51.8%, male = 46.3%, identified with another term = 1.7%, missing 0.2%). The number of participants who provided data eligible for analyses represented 82.5% of students enrolled across Bermudian private schools (*N* = 1495) and 57.8% of students enrolled across Government schools (*N* = 1031). The lower participation rate among students at public schools appeared to be the result of students declining to assent (12% Government compared to 3% private) rather than due being absent or lacking parental consent for the survey. A summary of the demographic characteristics of the sample is provided in Table 1.

### The prevalence of depression symptoms

Nearly one third (31.3%) of adolescents reported moderate to severe depression symptoms. Of the three independent variables examined (i.e. age, gender and race), all were significant: older adolescents, female gender and Black and Minority race (compared to White) were each associated with a higher prevalence of moderate to severe depression symptoms as compared to mild or no symptoms; AOR (older adolescents) = 1.474 (95% CI [1.226, 1.773)], *p* < 0 0.001), AOR (female) = 2.655 (95% CI [2.205, 3.196], *p* < 0.001), AOR (Black) = 1.791 (95% CI [1.419, 2.260], *p* < 0.001) and AOR (Minority) = 2.094 (95% CI [1.631, 2.687], *p* < 0.001).

Over one in five (22.6%) adolescents reported moderate to severe symptoms of depression and also functional impairment, indicating that these symptoms interfered with their lives in at least one of: daily activities, school or work, or in relationships with peers. Of the three independent variables examined, all were significant: older adolescents, female gender and Black and Minority race (compared to White) were each associated with a higher prevalence of moderate to severe depression symptoms with impairment as compared to moderate to severe depression symptoms with no impairment; AOR (older adolescents) = 1.465 (95% CI [1.201, 1.788], *p* < 0 0.001), AOR (female) = 2.645 (95% CI [2.150, 3.253], *p* < 0.001), AOR (Black) = 1.645 (95% CI [1.271, 2.129], *p* < 0.001) and AOR (Minority) = 1.675 (95% CI [1.268, 2.214], *p* < 0.001). Detailed results are shown in Table 2.

Among the eight depressive symptoms examined, the most commonly reported by adolescents was ‘Trouble falling or staying asleep, or sleeping too much’ (18.7%). The least common was ‘Moving or speaking so slowly that other people could have noticed, Or the opposite, being so fidgety or restless that you have been moving around a lot more than usual’ (6.2%). More detailed results are shown in Supplementary Table 1.

**Table 2:** Symptoms of depression among adolescents in Bermuda as assessed by PHQ-8

Variable		Age		Gender		Race		
	All participants (*N* = 2488)	Younger adolescents (*N* = 1590)	Older adolescents (*N* = 879)	Female (*N* = 1290)	Male (*N* = 1151)	Black (*N* = 1087)	White (*N* = 666)	Minority (*N* = 727)
Patient Health Questionnaire (PHQ-8)								
Mean score (± SD)	7.06 ± 5.79	6.54 ± 5.44	7.98 ± 6.25	8.24 ± 5.91	5.48 ± 5.06	7.24 ± 5.86	5.88 ± 5.40	7.85 ± 5.89
Depression severity, n (%)								
None - Minimal	1045 (38.5)	715 (41.9)	321 (33.3)	423 (30.7)	613 (48.3)	440 (36.6)	346 (49.8)	257 (32.3)
Mild	664 (24.4)	426 (24.9)	236 (24.5)	353 (25.7)	307 (24.2)	282 (23.4)	176 (25.3)	204 (25.7)
Moderate	461 (17.0)	287 (16.8)	170 (17.6)	300 (21.8)	151 (11.9)	230 (19.1)	80 (11.5)	148 (18.6)
Moderately severe	235 (8.7)	131 (7.7)	102 (10.6)	160 (11.6)	62 (4.9)	95 (7.9)	51 (7.3)	88 (11.1)
Severe	83 (3.1)	31 (1.8)	50 (5.2)	54 (3.9)	18 (1.4)	40 (3.3)	13 (1.9)	30 (3.8)
Missing	228 (8.4)	118 (6.9)	85 (8.8)	86 (6.3)	118 (9.3)	116 (9.6)	29 (4.2)	68 (8.6)
Presence depressive symptoms, n (%)								
Moderate to severe	779 (31.3)	**449 (28.2)**	322 (36.6)	514 (39.8)	**231 (20.1)**	365 (33.6)	**144 (21.6)**	266 (36.6)
Moderate to severe symptoms with impairment, n (%)								
Any impairment	562 (22.6)	**315 (19.9)**	242 (27.5)	375 (29.1)	**157 (13.6)**	274 (25.2)	**105 (15.8)**	181 (24.9)
Impairment in daily activities	371 (14.9)	194 (12.2)	175 (19.9)	249 (19.3)	100 (8.7)	180 (16.6)	75 (11.3)	116 (16.0)
Impairment in school or work	430 (17.3)	228 (14.3)	198 (22.5)	291 (22.6)	113 (9.8)	201 (18.5)	81 (12.2)	147 (20.2)
Impairment in relationships with peers	206 (8.3)	115 (7.2)	89 (10.1)	137 (10.6)	56 (4.9)	113 (10.4)	31 (4.7)	61 (8.4)

PHQ-8 Depression severity according to total score: None: 0–4, Mild: 5–9, Moderate: 10–14: Moderately-severe: 15–19, Severe: 20–24

Younger adolescents: 10–14 year olds; Older adolescents: 15–18 year olds

Impairment was considered present when participants reported that their symptoms ‘often’ or ‘always’ interfered with their functioning

Statistically significant effects are shown in bold

Older adolescents reported a higher prevalence of moderate to severe depression symptoms, with and without any impairment, compared to younger adolescents (*p* < 0.001)

Female adolescents reported a higher prevalence of moderate to severe depression symptoms, with and without any impairment, compared to male adolescents (*p* < 0.001)

Black and Minority adolescents reported a higher prevalence of moderate to severe depression symptoms, with and without any impairment, compared to White adolescents at (*p* < 0.001)

### The prevalence of anxiety symptoms

Over one in four (25.2%) of all adolescents reported experiencing moderate to severe anxiety symptoms. Of the three independent variables examined, all were significant except for race (Black): older adolescents, female gender and Minority race (compared to White) were each associated with a higher prevalence of moderate to severe anxiety symptoms as compared to mild or no symptoms; AOR (older adolescents) = 1.441 (95% CI [1.186, 1.751], *p* < 0 0.001), AOR (female) = 2.843 (95% CI [2.326, 3.476], *p* < 0.001) and AOR (Minority) = 1.418 (95% CI [1.098, 1.832], *p* < 0.01).

About one in five (19.1%) adolescents reported both moderate to severe symptoms of anxiety and also impairment with their lives in at least one of: daily activities, school or work, or relationships with peers. Of the three independent variables examined, all were significant except for race (Black): older adolescents, female gender and Minority race (compared to White) were each associated with a higher prevalence of moderate to severe anxiety symptoms with impairment as compared to moderate to severe anxiety symptoms with no impairment; AOR (older adolescents) = 1.459 (95% CI [1.178, 1.806], *p* < 0 0.001), AOR (female) = 2.742 (95% CI [2.188, 3.435], *p* < 0.001) and AOR (Minority) = 1.365 (95% CI [1.026, 1.814], *p* < 0.05. The detailed results are shown in Table 3.

Among the seven symptoms of anxiety examined, the most commonly reported was ‘Becoming easily annoyed or irritable’ (20.7%). The least common was ‘Being so restless that it is hard to sit still’ (8.4%). More detailed results are shown in Supplementary Table 2.

**Table 3:** Symptoms of anxiety among adolescents in Bermuda as assessed by GAD-7

Variable		Age		Gender		Race		
	All participants (*N* = 2502)	Younger adolescents (*N* = 1599)	Older adolescents (*N* = 882)	Female (*N* = 1297)	Male (*N* = 1157)	Black (*N* = 1095)	White (*N* = 672)	Minority (*N* = 726)
Generalised Anxiety Disorder-7 (GAD-7)								
Mean score (± SD)	6.17 ± 5.62	5.87 ± 5.40	6.72 ± 5.98	7.56 ± 5.82	4.41 ± 4.72	6.20 ± 5.58	5.45 ± 5.31	6.80 ± 5.91
Anxiety severity, n (%)								
None - Minimal	1201 (44.2)	794 (46.5)	395 (41.0)	480 (34.9)	710 (55.9)	510 (42.4)	365 (52.5)	321 (40.4)
Mild	670 (24.7)	440 (25.8)	227 (23.5)	388 (28.2)	273 (21.5)	314 (26.1)	161 (23.2)	194 (24.4)
Moderate	355 (13.1)	211 (12.4)	142 (14.7)	224 (16.3)	119 (9.4)	150 (12.5)	93 (13.4)	110 (13.8)
Severe	276 (10.2)	154 (9.0)	118 (12.2)	205 (14.9)	55 (4.3)	121 (10.1)	53 (7.6)	101 (12.7)
Missing	214 (7.9)	109 (6.4)	82 (8.5)	79 (5.7)	112 (8.8)	108 (9)	23 (3.3)	69 (8.7)
Presence anxiety symptoms, n (%)								
Moderate to severe	631 (25.2)	**365 (22.8)**	260 (29.5)	429 (33.1)	**174 (15.0)**	271 (24.7)	**146 (21.7)**	211 (29.1)
Moderate to severe symptoms with impairment, n (%)								
Any impairment	477 (19.1)	**271 (16.9)**	202 (22.9)	324 (25)	**126 (10.9)**	210 (19.2)	**109 (16.2)**	157 (21.6)
Impairment in daily activities	325 (13)	171 (10.7)	152 (17.2)	220 (17)	84 (7.3)	150 (13.7)	76 (11.3)	99 (13.6)
Impairment in school or work	371 (14.9)	199 (12.4)	168 (19.0)	261 (20.1)	87 (7.5)	155 (14.2)	84 (12.5)	131 (18)
Impairment in relationships with peers	180 (7.2)	102 (6.4)	77 (8.7)	121 (9.3)	47 (4.1)	88 (8)	38 (5.7)	54 (7.4)

GAD-7 Anxiety severity according to total score: None: 0–4, Mild: 5–9, Moderate: 10–14, Severe: 15–21

Impairment was considered present when participants reported that their symptoms ‘often’ or ‘always’ interfered with their functioning

Younger adolescents: 10–14 year olds; Older adolescents: 15–18 year olds

Statistically significant effects are shown in bold

Older adolescents reported a higher prevalence of moderate to severe anxiety symptoms, with and without any impairment, compared to younger adolescents (*p* < 0.001)

Female adolescents reported a higher prevalence of moderate to severe anxiety symptoms, with and without any impairment, compared to male adolescents (*p* < 0.001)

Minority adolescents reported a higher prevalence of moderate to severe depression symptoms, with any impairment (*p* < 0.05) and without impairment (*p* < 0.001), compared to White adolescents

### Comorbid symptoms of depression and anxiety

Almost one in five adolescents (19.9%) reported comorbid moderate to severe symptoms of depression and anxiety. The prevalence of moderate to severe comorbid anxiety symptoms in adolescents who reported moderate to severe depressive symptoms was 65.6%. Of the three independent variables examined, only gender was significant: female gender was associated with a higher prevalence of moderate to severe comorbid depression and anxiety symptoms compared to mild or no symptoms; AOR (female) = 1.916, 95% CI (1.382, 2.655), *p* < 0.001.

More than 80% (82.3%) adolescents reporting comorbid depression and anxiety symptoms also reported impairment. Of the three independent variables examined, none were significantly associated with a higher prevalence of comorbid depression and anxiety symptoms with impairment. The detailed results are shown in Table 4.

**Table 4:** The rate of comorbid symptoms of depression and anxiety among adolescents in Bermuda

Variable		Age		Gender		Race		
	All participants (*N* = 767)	Younger adolescents (*N* = 441)	Older adolescents (*N* = 318)	Female (*N* = 507)	Male (*N* = 226)	Black (*N* = 358)	White (*N* = 143)	Minority (*N* = 262)
Anxiety symptom severity among those experiencing moderate to severe depression symptoms, n (%)								
None	62 (8.1)	31 (7.0)	31 (9.7)	25 (4.9)	35 (15.5)	28 (7.8)	11 (7.7)	23 (8.8)
Mild	202 (26.3)	123 (27.9)	77 (24.2)	129 (25.4)	67 (29.6)	104 (29.1)	36 (25.2)	61 (23.3)
Moderate	257 (33.5)	149 (33.8)	106 (33.3)	172 (33.9)	75 (33.2)	119 (33.2)	53 (37.1)	83 (31.7)
Severe	246 (32.1)	138 (31.3)	104 (32.7)	181 (35.7)	49 (21.7)	107 (29.9)	43 (30.1)	95 (36.3)
Presence anxiety symptoms, n (%)								
Moderate to severe	503 (65.6)	287 (65.1)	210 (66.0)	353 (69.6)	**124 (54.9)**	226 (63.1)	96 (67.1)	178 (67.9)
Moderate to severe anxiety and depression symptoms with impairment, n (%)								
Any impairment	414 (82.3)	230 (80.1)	180 (85.7)	286 (81.0)	102 (82.3)	190 (84.1)	79 (82.3)	144 (80.9)
Impairment in daily activities	290 (57.7)	153 (53.3)	135 (64.3)	196 (55.5)	74 (59.7)	136 (60.2)	60 (62.5)	94 (52.8)
Impairment in school or work	328 (65.2)	170 (59.2)	154 (73.3)	233 (66.0)	72 (58.1)	142 (62.8)	63 (65.6)	122 (68.5)
Impairment in relationships with peers	154 (30.6)	84 (29.3)	69 (32.9)	107 (30.3)	35 (28.2)	80 (35.4)	25 (26.0)	49 (27.5)

All students who reported mild, moderate or severe symptoms of depression on the PHQ-8 were included

Impairment was considered present when participants reported that their symptoms ‘often’ or ‘always’ interfered with their functioning

Younger adolescents: 10–14 year olds; Older adolescents: 15–18 year olds

Statistically significant effects are shown in bold

Males reported significantly lower comorbid moderate to severe symptoms than Females at *p* < 0.001

## Discussion

This study is the first to estimate the prevalence of symptoms of depression and anxiety among adolescents aged 10–18 years in Bermuda. We estimate the prevalence of moderate to severe depressive and anxiety symptoms to be 31.3% and 25.2%, respectively. We also found that 65.6% of those reporting moderate to severe depression symptoms also reported comorbid moderate to severe anxiety symptoms.

Global averages for the prevalence of depression and anxiety symptoms among children and adolescents - median age 13.0 years– were estimated during the first year of the pandemic to be 25.5% and 20.5%, respectively [12]. This suggests that the estimated prevalence of depression and anxiety symptoms are higher in Bermuda than in other settings. The results for Bermuda, however, are in keeping with other findings from the Caribbean context, where studies have found relatively high rates of moderate to severe symptoms of depression (ranging 24.7% in St Kitts and Nevis to 40.7% in Jamaica) [4–8]. While none of the aforementioned studies reported impairment data, it is a matter of concern that respectively almost one in five (19.9%) and one in six (16.9%) adolescents were experiencing moderate to severe depression and anxiety symptoms with impairment in their daily activities, in school or work or in their relationships with peers.

With regards to age and gender differences, our results are largely in line with other studies: older adolescents reported significantly higher prevalence of depression and anxiety symptoms than younger adolescents, which is a finding that is supported by studies from the USA [39, 40]. Similarly, there was a higher prevalence reported among females than males, for both depression and anxiety and comorbid symptoms [12, 13, 41]. Limited local data is available to explain the gender differences in these symptoms in Bermuda; however, a wide range of causes have been suggested to explain this trend globally, including (but not limited to): the earlier onset of puberty, differences in coping strategies, increased sensitivity to distress in peers, and fear of rejection by peers [42–45].

Our findings regarding prevalence among different racial identity groups both supported and contradicted mixed findings from the USA. In many studies, there is a ‘Black-White paradox’ reported in the USA, suggesting that although Black persons are often found to have higher rates of physical illness, poverty and psychosocial stressors, they also report the same or better mental health than Whites persons [46–48, 21, 41]. In contrast, we found that Black adolescents reported a higher prevalence of depression symptoms than White adolescents, which is a finding that is reported elsewhere in the USA [22, 23]. On the other hand, White and Black adolescents reported a similar prevalence of moderate to severe anxiety symptoms and subsequent impairment, which supports the Black-White paradox [17] and other studies that show there is no difference between White and Black adolescents in the prevalence of diagnosed anxiety disorders [49]. It is questionable whether the conditions that lead to the Black-White paradox in the USA may apply to the Bermudian context. On one hand, unlike in the USA, Black Bermudians are not a racial minority in Bermuda, rather they are the racial majority and as such may not experience the same level of discrimination experiences as minority groups [50]. Discrimination among minority groups such as Black Americans has been reported to be associated with poorer mental health outcomes [51]. On the other hand, income inequality is high between racial groups. Black Bermudians ($59,099 USD) earn slightly more than those who identify as Mixed and Other ($56,327 USD), yet considerably less than White Bermudians ($89,302 USD); moreover, Black Bermudians make up 70% of the unemployed population compared to 15% White Bermudians and 15% Mixed and Other [16]. These findings are problematic as income inequality has been found to be positively associated with the prevalence of depression [52]. Further research is needed to determine what factors related to race contributing to the Black-White paradox in the USA, for example, higher rates of psychosocial stressors among Black Americans, are contributing to these mixed mental health findings in Bermuda. Nevertheless, these findings support the continued efforts to explain the Black-White paradox [53–55] as this may benefit our understanding of the driving factors of racial differences in the prevalence of depression symptoms among adolescents in Bermuda. A promising finding to explain the paradox in adolescents in the USA may be the higher levels of self-esteem found among Black adolescents compared to White adolescents [20]. As such, it would be useful for future research to determine whether the higher levels of self-esteem of Black adolescents in the USA extends to Black adolescents in the Caribbean. Adolescents that reported their race as Minority reported a high prevalence of moderate to severe depression symptoms and the highest prevalence of moderate to severe symptoms of anxiety. Given the relatively large Portuguese minority in Bermuda, our results may be seen to support the finding that Hispanic adolescents have a higher prevalence of depression symptoms as compared to other racial groups [56–58], and are significantly more likely to meet the clinical cut-off for social anxiety disorder compared to White and Black adolescents [59]. However, without further research it is difficult to determine what factors related to race can explain these findings. Indeed, racial differences in mental health, generally, and in Bermuda, reflect a complex interaction of biopsychosocial factors.

There is limited research that can provide specific insight to the driving factors for the relatively high rates of symptoms of depression and anxiety reported by adolescents in Bermuda. A recent scoping review of children and young people in the Caribbean reported that adverse events were consistently associated with mental health problems [60], which is consistent with a study on Adverse Childhood Experiences (ACEs) in Bermuda that highlighted the high prevalence of bullying and sexual and physical abuse, particularly among females [61]. As a growing body of literature show ACEs are risk factors for the development of adolescent depression and anxiety [62–67], future research investigating ACEs in adolescents may help explain the driving factors of the high prevalence of depression and anxiety symptoms in adolescents. Moreover, further research should investigate the help-seeking intentions, barriers and behaviours of adolescents in Bermuda. Understanding the nature of help-seeking among these adolescents would be instrumental in effective policy design and improving mental health service provision.

### Strengths and limitations

This study represents the first nationally representative survey of depression and anxiety symptoms in adolescents in Bermuda. The participation rate of over 70% of adolescents attending middle and high schools is high when considering the co-ordination required to get approval and participation of all eligible private and government-funded schools. Moreover, the current study contributes to bridging gaps in the coverage of prevalence data for adolescent mental health problems globally [3, 68–70]. In particular, our results provide new data for adolescents aged 10–11, which is lacking in the Caribbean [60], and makes a valuable contribution to our understanding of mental health problems among adolescents in the region [10].

This study has several key limitations. Self-report measures were used to screen for anxiety, depression and impairment, rather than a clinician-administered diagnostic interview such as the Composite International Diagnostic Interview (CIDI). This means that we are unable to estimate the prevalence of diagnosed depression and anxiety among adolescents in Bermuda. However, we used validated measures and aligned our data collection with contemporary global indicators for adolescent mental health [71] by also collecting data on impairment. It is possible that anxiety symptoms might have been underrepresented among certain subgroups, as the GAD-7 has been found to underestimate the prevalence of GAD in Black Americans [72]. There were lower participation rates in the senior public high schools compared to the private high schools and this means that our sample under-represented Black and older adolescents, and over-represented White, Minority and younger adolescents. However, the participation of all eligible middle and high schools meant a highly representative sample was obtained.

## Conclusions

Bermuda has a higher prevalence of moderate to severe symptoms of depression and anxiety among adolescents compared to global averages [12]. This study suggests that the prevalence of moderate to severe symptoms of depression, as well as the prevalence of these symptoms with impairment in functioning, were significantly more common among older adolescents, females and those who identified as Black or Minority. The prevalence of moderate to severe symptoms of anxiety was significantly more common among older adolescents, females and those who identified as Minority. Females and older adolescents reported significantly higher impairment with moderate to severe symptoms of anxiety. White adolescents reported significantly lower prevalence of moderate to severe symptoms of anxiety with impairment compared to Minority adolescents. Finally, males reported lower prevalence of moderate comorbid symptoms of depression and anxiety.

Given the high prevalence of symptoms of depression and anxiety among adolescents in Bermuda, this study suggests that greater policy attention and programming is needed to improve their mental health, including ongoing monitoring and evaluation of this vulnerable population. The demographic differences found across our adolescent sample in terms of the prevalence of the symptoms of depression and anxiety suggest that tailoring intervention programs would be advantageous. Older adolescents, females, and those who do not identify as White, stand to benefit greatly from such targeted interventions. Further research is warranted to understand the drivers of depression and anxiety symptoms and feasible interventions.

## Electronic supplementary material

Below is the link to the electronic supplementary material.

Supplementary Material 1

## Data Availability

Access to the data described within the manuscript and supplementary files can be granted upon an email request to the authors.
