# Supplementary material for: Prevalence estimates of depression and anxiety symptoms among adolescents in Bermuda, according to age, gender and race
Source: Soc Psychiatry Psychiatr Epidemiol. 2025 Mar 11;60(9):2189–200. doi: 10.1007/s00127-025-02829-z (PMC12378848; doi:10.1007/s00127-025-02829-z)
Supplement: Supplementary file 1 — Supplementary Material 1 [file 127_2025_2829_MOESM1_ESM.docx]

| Supplementary Table 1: The presence of PHQ-8 symptoms by age, gender and race | | |  |  |  |  |  |  |  |
| --- | --- | --- | --- | --- | --- | --- | --- | --- | --- |
| Variable |  | Age | | Gender | | Race | | |  |
|  | All | Younger adolescents | Older adolescents | Female | Male | Black | White | Minorities |  |
|  | (N = 2488) | (N = 1590) | (N = 879) | (N = 1290) | (N = 1151) | (N = 1087) | (N = 666) | (N = 727) |  |
| PHQ items, n (%) |  |  |  |  |  |  |  |  |  |
| Little interest or pleasure in doing things. | 260 (10.5) | 152 (9.6) | 102 (11.6) | 148 (11.5) | 97 (8.4) | 133 (12.2) | 43 (6.5) | 83 (11.4) |  |
| Feeling down, depressed, or hopeless. | 184 (7.4) | 98 (6.2) | 85 (9.7) | 107 (8.3) | 65 (5.6) | 86 (7.9) | 32 (4.8) | 66 (9.1) |  |
| Trouble falling or staying asleep, or sleeping too much. | 466 (18.7) | 277 (17.4) | 185 (21.0) | 297 (23) | 146 (12.7) | 212 (19.5) | 99 (14.9) | 155 (21.3) |  |
| Poor appetite, weight loss, or overeating. | 255 (10.2) | 131 (8.2) | 121 (13.8) | 173 (13.4) | 67 (5.8) | 118 (10.9) | 36 (5.4) | 100 (13.8) |  |
| Feeling tired or having little energy. | 431 (17.3) | 228 (14.3) | 200 (22.8) | 274 (21.2) | 139 (12.1) | 198 (18.2) | 96 (14.4) | 136 (18.7) |  |
| Feeling bad about yourself – or that you are a failure or have let yourself or your family down. | 318 (12.8) | 186 (11.7) | 129 (14.7) | 216 (16.7) | 84 (7.3) | 153 (14.1) | 50 (7.5) | 113 (15.5) |  |
| Trouble concentrating on things like school work, reading or watching TV? | 375 (15.1) | 207 (13.0) | 165 (18.8) | 227 (17.6) | 124 (10.8) | 171 (15.7) | 89 (13.4) | 114 (15.7) |  |
| Moving or speaking so slowly that other people could have noticed? Or the opposite, being so fidgety or restless that you have been moving around a lot more than usual? | 154 (6.2) | 96 (6.0) | 55 (6.3) | 89 (6.9) | 54 (4.7) | 78 (7.2) | 24 (3.6) | 52 (7.2) |  |

| Supplementary Table 2: The presence of GAD-7 symptoms by age, gender and race | | |  |  |  |  |  |  |
| --- | --- | --- | --- | --- | --- | --- | --- | --- |
| Variable |  | Age | | Gender | | Race | | |
|  | All | Younger adolescents | Older adolescents | Female | Male | Black | White | Minorities |
|  | (N = 2502) | (N = 1599) | (N = 882) | (N = 1297) | (N = 1157) | (N = 1095) | (N = 672) | (N = 726) |
| GAD-7 items, n (%) |  |  |  |  |  |  |  |  |
| Feeling nervous, anxious or on edge. | 288 (11.5) | 148 (9.3) | 137 (15.5) | 208 (16) | 64 (5.5) | 119 (10.9) | 79 (11.8) | 89 (12.3) |
| Not being able to stop or control worrying. | 279 (11.2) | 149 (9.3) | 129 (14.6) | 202 (15.6) | 62 (5.4) | 117 (10.7) | 66 (9.8) | 96 (13.2) |
| Worrying too much about different things. | 387 (15.5) | 207 (12.9) | 177 (20.1) | 274 (21.1) | 97 (8.4) | 166 (15.2) | 90 (13.4) | 131 (18) |
| Trouble relaxing. | 213 (8.5) | 126 (7.9) | 85 (9.6) | 127 (9.8) | 71 (6.1) | 84 (7.7) | 41 (6.1) | 87 (12) |
| Being so restless that it is hard to sit still. | 209 (8.4) | 134 (8.4) | 71 (8.0) | 127 (9.8) | 70 (6.1) | 92 (8.4) | 39 (5.8) | 78 (10.7) |
| Becoming easily annoyed or irritable. | 517 (20.7) | 328 (20.5) | 184 (20.9) | 346 (26.7) | 145 (12.5) | 256 (23.4) | 88 (13.1) | 171 (23.6) |
| Feeling afraid as if something awful might happen. | 324 (12.9) | 203 (12.7) | 119 (13.5) | 220 (17) | 90 (7.8) | 158 (14.4) | 51 (7.6) | 114 (15.7) |
